# Discrete Element Modeling of the Meso-Mechanical Response of Asphalt Pavement under Vehicle Load

**DOI:** 10.3390/ma15217808

**Published:** 2022-11-05

**Authors:** Dong Zhang, Chunying Wu, Lili Cai, Jiang Bian, Chaoen Yin

**Affiliations:** 1 College of Transportation Engineering, Nanjing Tech University, Nanjing 211816, China; 2 Nanjing Ningtong Intelligent Transportation Technology Research Institute Co., Ltd., Nanjing 211135, China

**Keywords:** asphalt pavement, discrete element method, meso-mechanical response, contact forces

## Abstract

Numerical simulation is an effective way to study the mechanical response of asphalt pavement, which is very important for the pavement structural design. In this study, a three-dimensional meso-structure discrete element model of asphalt pavement was generated with the FISH programming language and its meso-mechanical response under vehicle load was analyzed. The contact forces within the asphalt pavement, in asphalt mastic, in coarse aggregates and between asphalt mastic and coarse aggregates were studied. The results of the study show that the contact forces within the asphalt mixture are highly uneven. The number of contact points in coarse aggregates account only for about 10% of all contact points while the sum of the contact forces in coarse aggregates contributes to over 50% of all contact forces. This demonstrates that the coarse aggregates bear most of the vehicle load. The average normal contact force in coarse aggregates is about 5 N and the average tangential contact force in coarse aggregates is about 2 N. The modeling results provide a quantitative understanding of the distribution of loading in asphalt pavement.

## 1. Introduction

Vehicle load is the main load for asphalt pavement. Several pavement distresses like rutting and cracking are caused by repeated vehicle load action. A numerical simulation is an effective method to study the failure mechanism in asphalt pavement. In previous studies, the finite element method (FEM) and discrete element method (DEM) have been widely used to study the mechanical response of asphalt pavement under vehicle load.

Gungor et al. [1] proposed a three-dimensional finite element model to simulate the inter-action of the tire and asphalt pavement. Assogba et al. [2] studied the dynamic mechanical response of asphalt pavement at different depths by the finite element software. Wang et al. [3] developed a finite element model to simulate the mechanical response of asphalt pavement under the multiple wheel load by considering the constitutive models of road materials. Zhang et al. [4] and Lu et al. [5] studied the mechanical response of asphalt pavement by employing a nonlinear anisotropic elastoplastic constitutive model of the base material.

The FEM is a practical method in the analysis of the macroscopic mechanical response of asphalt pavement. In asphalt pavement, the response in asphalt layers is the research emphasis. The asphalt mixture is generally considered to be a homogeneous and continuous material in the FEM model. However, the asphalt mixture is a multi-phase composite material which is mainly composed of aggregate particles, asphalt mastic and voids [6,7,8]. In the asphalt mixture, coarse aggregate particles and asphalt mastic differ greatly from each other with respect of mechanical parameters. The mechanical properties of the asphalt mixture in various directions are also affected by the distribution and orientation of the coarse aggregate particles [9,10,11,12]. Therefore, the FEM cannot truly simulate the mechanical response in the asphalt mixture.

In recent years, increasing attentions have been gained to model the actual meso-structure of asphalt mixtures and the DEM has been employed to study the meso-mechanical response of asphalt pavement. Unlike the FEM, the DEM uses simple constitutive models at contacts between particles to model complicated mechanical behaviors of materials [13,14]. It is potentially more effective to simulate the meso-mechanical response in asphalt mixtures. For the discrete element simulation of asphalt mixtures, two categories of research work have been carried out. One is the reconstruction of meso-structure of asphalt mixtures with the DEM. The other is the discrete element simulation of the mechanical response in asphalt mixtures.

The image analysis method and the X-ray computed tomography technology were used to generate the two-dimensional and three-dimensional discrete element samples of asphalt mixtures [15,16,17,18]. Wang et al. [19,20] and Fu et al. [21] developed algorithms to generate the three-dimensional aggregates and granular mixtures in the discrete element software with the digital data of aggregates. The digital data were collected by the X-ray computed tomography technology. This method is costly and time-consuming due to that it relies on expensive devices. The random generation algorithms were proposed to solve this problem. Lu and McDowell [22] proposed an algorithm to model the granular particle using overlapping balls. The sizes and location of the particles can be adjusted by the random algorithm in their studies. Zhang et al. [23] proposed a random algorithm to generate the three-dimensional aggregate particles and asphalt mixture samples in the discrete element software. The algorithm can generate aggregate particles with different shapes, sizes and angularity.

With the DEM, the mechanical response of asphalt mixtures can be simulated. You and Buttlar [18,24,25] studied the dynamic modulus and creep stiffness of asphalt mixtures by the DEM. Liu et al. [16] and You et al. [26] developed the viscoelastic discrete element model for asphalt mixtures and simulated the creep compliance tests of asphalt mixtures. Kim et al. [27,28,29] embedded the cohesive zone model into the discrete element samples of asphalt mixtures and studied the fracture behavior in the asphalt mixture specimens. Zhang et al. [30] studied the micro-cracking behavior of asphalt mixtures in the indirect tensile test with the DEM. Ma et al. [31] simulated the wheel tracking test of asphalt mixtures with a three-dimensional micromechanical discrete element model and studied the movements of coarse aggregates during the test. Peng et al. [32] developed a three-dimensional discrete element model of asphalt pavement by considering the temperature gradient and fatigue damage and studied the permanent deformation, shear stress and strain in asphalt surfaces under moving traffic load.

The objective of this study is to investigate the meso-mechanical response in asphalt pavement under vehicle load by the discrete element modeling. To achieve this goal, a three-dimensional meso-structure discrete element model of asphalt pavement was generated with the FISH programming language and the contact forces within the asphalt pavement, in asphalt mastic, in coarse aggregates and between asphalt mastic and coarse aggregates were studied. The study results contribute to a quantitative understanding of the distribution of vehicle load in asphalt pavement from a meso-mechanical level.

## 2. Discrete Element Model

### 2.1. Reconstruction of Asphalt Mixture Meso-Structure

**Figure 1:**
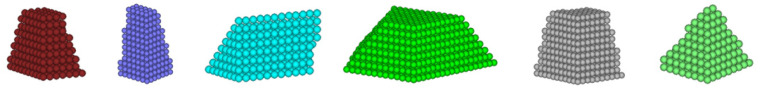
Coarse aggregates generated with the algorithm.

In previous research, the authors proposed a discrete element algorithm for generating three-dimensional meso-structure of asphalt mixtures [23]. The main steps are as follows: (1) generate the coarse aggregates with balls of identical radius (as shown in Figure 1) and place the coarse aggregates randomly in the sample; (2) generate the asphalt mastic which is simulated with regularly arranged balls; (3) delete a certain number of asphalt mastic balls between the asphalt mastic and coarse aggregates to model the air voids. This algorithm was employed to generate the discrete element model of asphalt mixture in this study.

### 2.2. Pavement Structure and Parameters

Taking the asphalt layers as the research object, the other parts of the pavement were simplified as wall. The pavement structure combination and parameters are shown in Table 1. The bulk density of the asphalt mixture is 2.4 g/cm^3^ and the apparent density of the coarse aggregates is 2.8 g/cm^3^ [23,30]. In order to reduce the number of balls in the discrete element model, coarse aggregate particles were constructed using balls with a radius of 2 mm. Aggregates with grain size of 4.75 mm or more were regarded as coarse aggregates. The asphalt mastic was assumed to be a combination of the fine aggregates passing the 4.75 mm sieve, fines and asphalt binder. The gradation of the coarse aggregates in AC-13, AC-20 and AC-25 is determined according to Chinese “Technical Specifications for Construction of Highway Asphalt Pavements (JTG F40-2004)”, as shown in Table 2.

In the discrete element simulation, the parallel-bond model was used for the bonds in coarse aggregates, in asphalt mastic and between asphalt mastic and coarse aggregates. The elastic modulus of coarse aggregates was set to be 55 GPa [23,30]. According to the laboratory test results at 15 °C, the elastic modulus of the asphalt mastic is 500 MPa and the tensile strength of the asphalt mastic is 1.2 MPa. The mesoscopic parameters of the balls in coarse aggregates, balls in the asphalt mastic and the parallel-bond model are calculated according to the conversion relationship between the mesoscopic parameters and the macroscopic parameters [23,30]. The calculation results are shown in Table 3.

### 2.3. Load, Model Dimensions and Boundary Conditions

**Figure 2:**
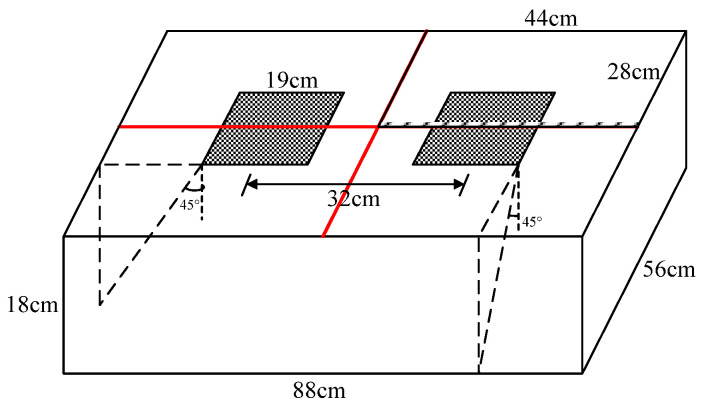
Load area and model dimensions.

**Figure 3:**
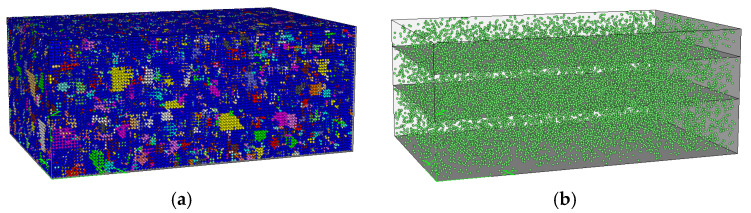
Discrete element model of asphalt pavement. (**a**) Discrete element model. (**b**) Voids distribution in the model.

According to Chinese “Specifications for Design of Highway Asphalt Pavement (JTG D50-2006)”, the standard axle load is a single axle with double wheel which weighs 100 kN (BZZ-100). The grounding pressure of the tire is 0.7 MPa with the equivalent circle radius of 10.65 cm. The center distance of the two circles is about 32 cm. According to the principle of equal area, the circular load is converted into a square load with a side length of about 19 cm. In order to reduce the number of particles in the model, it is necessary to reduce the sizes of the model as much as possible. However, the sizes of the model cannot be smaller than the area affected by the wheel load. This study introduces the concept of load spread angle in solid mechanics to determine the range of influence of standard axle load. For the sake of safety, the spread angle of load in asphalt mixture is taken as 45° and the model sizes determined thereby are shown in Figure 2. According to this, the number of particles is still large. In order to further reduce the sizes of the model, 1/4 of the complete model is studied according to the principle of axial symmetry, as shown in the shaded part in Figure 2. Figure 3 shows the discrete element model of asphalt pavement established in this paper. Figure 3b shows the voids distribution in the model. The voids were modeled by randomly deleting a certain number of asphalt mastic balls between the asphalt mastic and coarse aggregates according to the air void rate of asphalt mixtures.

In order to simulate the standard axle load, regularly arranged interconnected balls with a radius of 2 mm were generated. A vertical downward force of 11.2 N was then applied to each ball to simulate the grounding pressure of 0.7 MPa.

The boundary conditions of the model are as follows: (1) the walls were placed at the bottom and the periphery of the model to simulate the binding effect of the base layer and the asphalt mixture around the model; (2) the wall on the top of the model was deleted and a vertical force of 11.2 N was applied downward to each ball within the wheel load range. The translational and rotational movement speed of the balls within the wheel load range was fixed to zero in both the X and Y directions.

## 3. Results and Discussion

**Figure 4:**
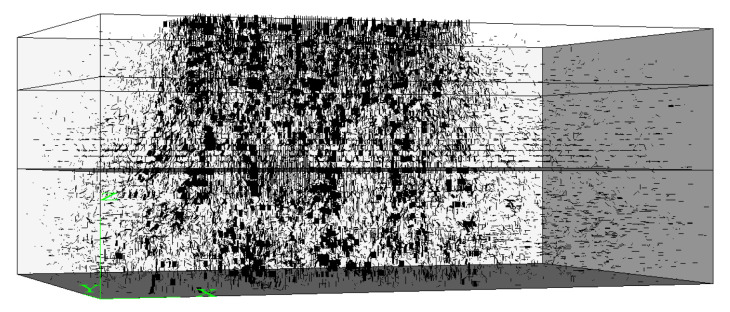
Distribution of the contact forces.

Apply the standard axle load to the model and run the program until the model reaches equilibrium. Figure 4 shows the distribution of contact forces in the discrete element model of asphalt pavement. The contact forces are indicated by short black lines. The thicker the line, the greater the contact force. It can be seen from Figure 4 that: (1) under wheel load, the contact forces in asphalt pavement are mainly distributed under the wheel; (2) from the upper layer to the bottom layer, the forces gradually spread from the load area to a broader area and the spread angle is small; (3) from the perspective of Figure 4, the upper, the middle and the lower layer of the asphalt pavement bear the wheel load to the same extent. The following sections will quantitatively study the distribution of the normal and the tangential contact forces in each layer of the asphalt pavement.

**Figure 5:**
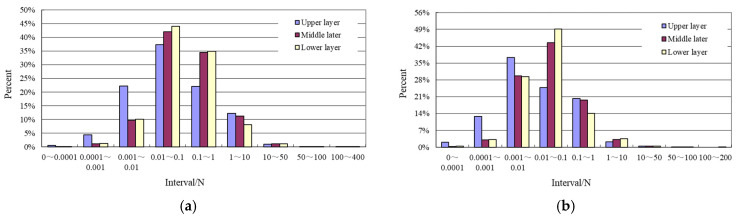
Diagram of distribution frequency of the contact forces in each asphalt layer. (**a**) Normal contact force. (**b**) Tangential contact force.

The normal contact forces and the tangential contact forces at the contact points in the asphalt mixture are extracted by scanning the results from the simulation. Table 4 shows the maximum contact force in each layer. As can be seen from Table 4:

(1) Under the standard axle load, the maximum normal contact force in coarse aggregates in the upper layer can reach 378 N and in the middle and lower layer this value can reach 280 N. The maximum tangential contact force in coarse aggregates in the upper layer can reach 121 N and in the middle and lower layer this value is 110 N. Hou et al. [33] studied the micro-mechanical response of asphalt mixtures by the DEM. In their study, a reduced model with a length of 15 cm, a width of 15 cm and a thickness of 6 cm was used and the load area in the model was reduced proportionally. The maximum contact force is between 80 N and 90 N within the model under the vehicle load of 0.7 Mpa. This demonstrates that simulation with reduced models may give wrong calculation results. Full-scale model is necessary in the discrete element simulation.
(2) The maximum normal contact force and the maximum tangential contact force in asphalt mastic and between asphalt mastic and coarse aggregates are at the same magnitude order. The maximum normal contact force is one order of magnitude lower than the maximum contact force in coarse aggregates. The maximum tangential contact force is two orders of magnitude lower than that value in coarse aggregates. The maximum normal contact force and the maximum tangential contact force between coarse aggregates and asphalt mastic in each layer are, respectively, about twice the maximum normal contact force and the maximum tangential contact force in asphalt mastic.
(3) As can be seen from Figure 5, most of the contact forces are lower than 10 N. The percent of the normal contact forces that exceed 10 N is between 1.0% and 1.4% in each layer. The percent of the tangential contact forces that exceed 10 N is between 0.4% and 0.6%.

Figure 5 shows the distribution of contact forces within each asphalt layer. As shown in Figure 5:

(1) The normal and tangential contact forces in the middle and the lower layer have similar distribution patterns. The normal contact forces are mainly distributed in the four intervals of (10^−3^ N, 10^−2^ N], (10^−2^ N, 10^−1^ N], (0.1 N, 1 N] and (1 N, 10 N). More than 75% of the normal contact forces lie in the interval (10^−2^ N, 10^−1^ N] and (0.1 N, 1 N). The tangential contact forces are mainly distributed in the intervals of (10^−3^ N, 10^−2^ N], (10^−2^ N, 10^−1^ N] and (0.1 N, 1 N). More than 42% of the tangential contact forces lie in the interval (10^−2^ N, 10^−1^ N).
(2) The upper layer has more contact forces in the smaller value range than the middle and the lower layer. The normal contact forces are mainly distributed in the four intervals of (10^−3^ N, 10^−2^ N], (10^−2^ N, 10^−1^ N], (0.1 N, 1 N] and (1 N, 10 N). The normal contact forces that in the interval (10^−2^ N, 10^−1^ N) occupy 37% of all contact forces in the upper layer. The tangential contact forces are mainly distributed in the interval (10^−4^ N, 10^−3^ N], (10^−3^ N, 10^−2^ N], (10^−2^ N, 10^−1^ N] and (0.1 N, 1 N), with (10^−2^ N, 10^−1^ N) the highest.

**Figure 6:**
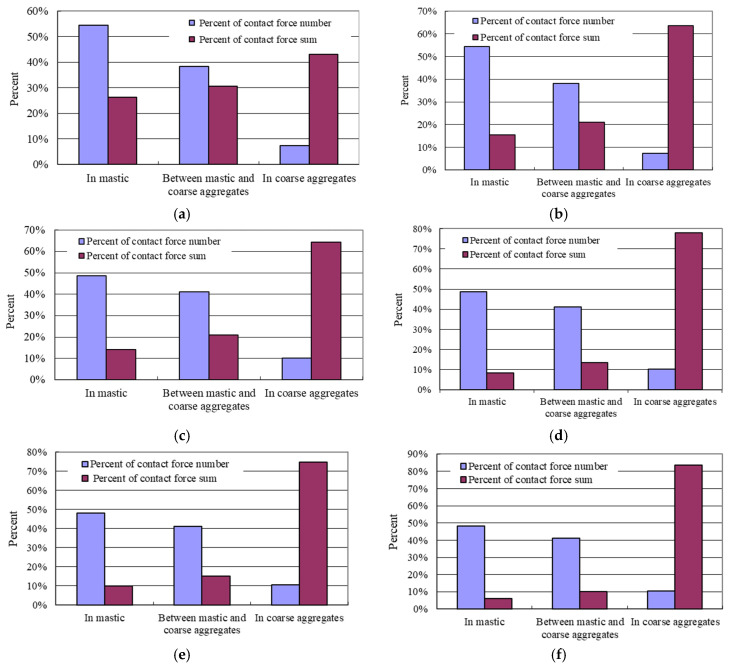
Percent of the contact forces in the asphalt mixture of each layer. (**a**) Normal contact force in the upper layer. (**b**) Tangential contact force in the upper layer. (**c**) Normal contact force in the middle layer. (**d**) Tangential contact force in the middle layer. (**e**) Normal contact force in the lower layer. (**f**) Tangential contact force in the lower layer.

Figure 6 shows the relative number and the sum of contact forces in percentage in asphalt mastic, between coarse aggregates and asphalt mastic and in coarse aggregates. As can be seen from Figure 6:

(1) In each layer, the number of contact forces in asphalt mastic accounts for about 50%, the number of contact forces between coarse aggregates and asphalt mastic accounts for about 40%, and the number of contact forces in coarse aggregates accounts for only about 10%.
(2) In the upper layer, the sum of the normal contact forces in coarse aggregates accounts for about 43% and the sum of the tangential contact forces accounts for about 64%. In the middle layer, the sum of the normal contact forces in coarse aggregates is about 64% and the sum of the tangential contact forces is about 78%. In the lower layer, the sum of the normal contact forces in coarse aggregates is about 75% and the sum of the tangential contact forces in coarse aggregates is about 84%.
(3) The above data indicates that the wheel load transmitted to the asphalt surface layer is mainly born by the coarse aggregates in each layer. A similar conclusion was also got by the study of Hou et al. [33]. Since the proportion of coarse aggregates in the AC-13 mixture of the upper layer is relatively low and AC-13 has a typical suspended-dense structure, the percent of the normal contact forces and the tangential contact forces in coarse aggregates in the upper layer is lower than that in the middle and the lower layers. Compared to the study of Hou et al. [33], this study further reveals the bearing characteristics of asphalt pavement due to that a three-layer asphalt pavement model was used in the simulation.

**Figure 7:**
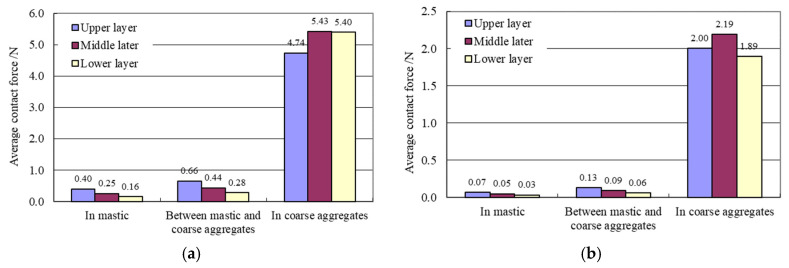
Average of the contact forces in each asphalt layer. (**a**) Normal contact force. (**b**) Tangential contact force.

Figure 7 shows the average of the contact forces in asphalt mastic, in coarse aggregates and between asphalt mastic and coarse aggregates. It can be seen from Figure 7 that:

(1) Under the standard axle load, the average normal contact force in coarse aggregates in all layers is about 5 N and the average tangential contact force is about 2 N. Ma et al. [31] simulated the wheel tracking test of asphalt mixtures with a three-dimensional micromechanical discrete element model. In their study, the average contact force within the aggregate skeleton was monitored. The results show that the average contact force is between 1.5 N and 2 N which is smaller than that computed by this study. The difference is caused by the model sizes and loading area. The average normal contact force between coarse aggregates and asphalt mastic in all layers lies between 0.2 N and 0.7 N and the average tangential contact force is between 0.05 N and 0.15 N. The average normal contact force in asphalt mastic in all layers is between 0.1 N and 0.5 N and the average tangential contact force is between 0.03 N and 0.07 N.
(2) In each layer, the average normal contact force and the average tangential contact force in coarse aggregates are much larger than those in asphalt mastic and between coarse aggregates and asphalt mastic. This further proves that coarse aggregates are the main load bearing component in each layer.
(3) The average normal contact force in coarse aggregates show very little difference among the upper, the middle and the lower layer. So are the average tangential contact force. This shows that all three layers of the asphalt pavement are the main bearers of the wheel load. This is consistent with the conclusions obtained from Figure 4.
(4) In each layer, the average contact force at different positions is significantly different. This proves that the internal contact forces of the asphalt mixture under load are highly uneven.

## 4. Conclusions

This study investigates the meso-mechanical response of asphalt pavement under vehicle load by the discrete element method. A three-layer asphalt pavement discrete element model was generated with the FISH programming language. The contact forces within the asphalt pavement, in asphalt mastic, in coarse aggregates and between asphalt mastic and coarse aggregates were studied. The statistical analysis of contact forces within each asphalt layer shows that the contact forces within the asphalt mixture are highly uneven and the coarse aggregates bear most of the vehicle load. The average normal contact force in coarse aggregates is about 5 N and the average tangential contact force in coarse aggregates is about 2 N.

One limitation of this study is that the meso-mechanical response of asphalt pavement was obtained at room temperature and under static load. In actual asphalt pavement, the temperature varies, and the vehicle load moves with time. Effect of temperature and moving load on the meso-mechanical response will be considered in the future study. Additionally, the simulation results in this study were not verified by laboratory or field tests due to that the meso-mechanical contact forces cannot be measured with existing technology. In spite of these limitations, this study contributes to a quantitative understanding of the distribution of vehicle load in asphalt pavement from a meso-mechanical level.

## Figures and Tables

**Table 1:** Pavement structure combination and parameters.

Layer Position	Asphalt Mixture Type	Thickness/cm	Air Void Rate/%	Asphalt Content/%
Upper layer	AC-13	4	3	6
Middle layer	AC-20	6	4	5
Lower layer	AC-25	8	5	5

**Table 2:** Gradation of coarse aggregates in AC-13, AC-20 and AC-25.

Sieve Size/mm		31.5	26.5	19	16	13.2	9.5	4.75
Passing rate/%	AC-13	100	100	100	100	95	76.5	53
	AC-20	100	100	95	85	71	61	41
	AC-25	100	95	82.5	74	66.5	55	38

**Table 3:** Mesoscopic parameters of the balls and parallel-bond model.

Category	Balls in Coarse Aggregates		Balls in Asphalt Mastic		Parallel-Bond Model				
Parameter	kn	ks	kn	ks	pb_kn	pb_ks	pb_nstr	pb_sstr	pb_radius
Unit	N/m	N/m	N/m	N/m	N/m^3^	N/m^3^	Pa	Pa	mm
Value	4.4 × 10^8^	2.0 × 10^8^	4.0 × 10^6^	1.33 × 10^6^	2.5 × 10^11^	2.34 × 10^10^	1.2 × 10^6^	1.2 × 10^6^	0.5

**Table 4:** Maximum contact force in asphalt surface layers.

Position	Values (N)					
	In Normal Direction			In Tangential Direction		
	In Mastic	Between Coarse Aggregates and Mastic	In Coarse Aggregates	In Mastic	Between Coarse Aggregates and Mastic	In Coarse Aggregates
Upper layer	14.9	32.6	378	3.34	5.13	121
Middle later	10.8	18.3	282	2.1	4.99	106
Lower layer	7.06	17	280	1.87	2.67	113

## Data Availability

The data presented in this study are available from the corresponding author upon reasonable request.
